# Polymeric Nanoparticle-Based Photodynamic Therapy for Chronic Periodontitis *in Vivo*

**DOI:** 10.3390/ijms17050769

**Published:** 2016-05-20

**Authors:** Laura Marise de Freitas, Giovana Maria Fioramonti Calixto, Marlus Chorilli, Juçaíra Stella M. Giusti, Vanderlei Salvador Bagnato, Nikolaos S. Soukos, Mansoor M. Amiji, Carla Raquel Fontana

**Affiliations:** 1 Faculdade de Ciencias Farmaceuticas, UNESP—Univ Estadual Paulista, Campus Araraquara, Departamento de Análises Clínicas, Araraquara, SP 14800-903, Brazil; lfmarise@gmail.com; 2 Faculdade de Ciencias Farmaceuticas, UNESP—Univ Estadual Paulista, Campus Araraquara, Departamento de Farmacos e Medicamentos, Araraquara, SP 14800-903, Brazil; giovana.calixto@gmail.com (G.M.F.C.); chorilli@fcfar.unesp.br (M.C.); 3 Instituto de Fisica de Sao Carlos, Universidade de Sao Paulo, Caixa Postal 369, Sao Carlos, SP 15980-900, Brazil; jsmgiusti@gmail.com (J.S.M.G.); vander@ifsc.usp.br (V.S.B.); 4 Applied Molecular Photomedicine Laboratory, the Forsyth Institute, 245 First Street, Cambridge, MA 02142, USA; n.soukos@neu.edu; 5 Department of PharmaceuticalSciences, School of Pharmacy, Bouvé College of Health Sciences, Northeastern University, 140 The Fenway, Room 156, 360 Huntington Avenue Boston, MA 02115, USA; m.amiji@neu.edu

**Keywords:** nanoparticles, PLGA, photodynamic therapy, periodontitis, methylene blue, biofilms

## Abstract

Antimicrobial photodynamic therapy (aPDT) is increasingly being explored for treatment of periodontitis. Here, we investigated the effect of aPDT on human dental plaque bacteria in suspensions and biofilms *in vitro* using methylene blue (MB)-loaded poly(lactic-*co*-glycolic) (PLGA) nanoparticles (MB-NP) and red light at 660 nm. The effect of MB-NP-based aPDT was also evaluated in a clinical pilot study with 10 adult human subjects with chronic periodontitis. Dental plaque samples from human subjects were exposed to aPDT—in planktonic and biofilm phases—with MB or MB-NP (25 µg/mL) at 20 J/cm^2^ *in vitro*. Patients were treated either with ultrasonic scaling and scaling and root planing (US + SRP) or ultrasonic scaling + SRP + aPDT with MB-NP (25 µg/mL and 20 J/cm^2^) in a split-mouth design. In biofilms, MB-NP eliminated approximately 25% more bacteria than free MB. The clinical study demonstrated the safety of aPDT. Both groups showed similar improvements of clinical parameters one month following treatments. However, at three months ultrasonic SRP + aPDT showed a greater effect (28.82%) on gingival bleeding index (GBI) compared to ultrasonic SRP. The utilization of PLGA nanoparticles encapsulated with MB may be a promising adjunct in antimicrobial periodontal treatment.

## 1. Introduction

Periodontitis is an inflammatory disease of the supporting tissues of the teeth caused by bacterial infection which, if left untreated, can eventually lead to tooth loss [1]. Tissue destruction occurs as a consequence of the host immune inflammatory response to oral pathogens [2]. Hundreds of different bacterial species have been isolated from periodontal pockets, and a subset of a selected number of species has been associated with periodontitis [3]. Those species participate in the formation of a biofilm on subgingival tooth surfaces in an interdependent form, where early colonizers pose as substrate for the adherence of species that will act as bridges between those early and the late colonizers [4].

Mechanical removal of the periodontal biofilms (scaling and root planing—SRP) remains the cornerstone of periodontal therapy. Antibiotics are also used, but they are associated with resistance concerns and poor efficacy against biofilms [5]. Another problem associated with the use of antibiotics in periodontitis is the difficulty to achieve bactericidal concentrations of the drug in the gingival crevicular fluid, for the complete eradication of pathogens [6].

One of the most promising approaches to overcome the above-mentioned drawbacks is the use of antimicrobial photodynamic therapy (aPDT). In aPDT, a photoactivated compound or photosensitizer (PS), which has been taken up by microorganisms, is activated by visible light of a specific wavelength to produce reactive oxygen species (ROS), including highly cytotoxic singlet oxygen [7]. The main advantages of aPDT over conventional antimicrobial therapies include the immediate onset of action, elimination of resistant microorganisms and secreted virulence factors, local delivery of PS, and double selectivity (deleterious effect only on sites where both PS and light are delivered concomitantly) [8]. The adjunctive use of aPDT in the treatment of periodontitis has been suggested as an alternative to chemical antimicrobial agents for elimination of subgingival species [9]. However, although it has been proven effective in a few clinical trials [10,11,12,13], aPDT beneficial effects over SRP alone are not evident in single sessions, as supported by a recent meta-analysis [14].

Several studies have shown that oral bacteria in planktonic cultures [15,16] and in plaque scrapings [15,17] are susceptible to PDT. However, our previous study [18] has shown incomplete eradication of oral bacteria in biofilms—following aPDT. Several other studies have also shown incomplete destruction of oral biofilms using MB-mediated aPDT [19,20]. Biofilms exhibit reduced susceptibility not only to PDT but to antimicrobial treatments in general, which is attributed to reduced penetration of the PS and other drugs deep in the biofilm matrix [21]. In addition, it has been shown that MB and other phenothiazine derivatives are substrates of multidrug resistance efflux pumps in bacteria, which decreases the effectiveness of the few PS molecules that are able to penetrate the biofilm matrix [22]. Such drawbacks can be overcome by the development of drug delivery systems such as nanoparticles (NPs), which significantly improve the pharmacological characteristics of the PS, e.g., increased local retention times, improved solubility and absorption, and protection against degradation and/or efflux [23,24,25,26].

The physicochemical features of nanoparticles, such as ultra-small size, concomitantly large surface to mass ratio, and highly reactive surface, confer numerous advantages for drug delivery, especially controlling the physicochemical behavior of the drug (solubility and release), and drug targeting to the potential active site, decreasing adverse effects [27,28,29,30,31]. Regarding PDT, nanoparticles containing PS carry several advantages over free PS. These advantages include [32,33]: (1) a larger critical mass (concentrated package of PS) for the local production of ROS; (2) limit the target cell’s ability to pump the drug back out, thus reducing the possibility of multidrug resistance; (3) increase treatment selectivity by the localized delivery of agents, which can be achieved by either passive or active targeting; and (4) the nanoparticle matrix is non-immunogenic.

Among a wide variety of NPs, polymer-based ones display a few advantages regarding drug delivery and have gained interest in PDT studies recently [34,35]. Two of the most remarkable properties of polymeric NPs are biocompatibility and low toxicity, besides their easy and straightforward process to fabricate stable formulations [27,36]. The most widely used polymeric NPs to date are those composed of poly(lactic-*co*-glycolic acid) (PLGA). PLGA’s physicochemical properties, biodegradation rate, and *in vivo* behavior can be modified by manipulating molecular weight, lactic acid:glycolic acid ratio, and end group [37]. The biodegradable property of PLGA is due to the products of its hydrolysis, lactic acid and glycolic acid, two endogenous and easily metabolized monomers, resulting in a minimal systemic toxicity associated with the use of PLGA for drug delivery or biomaterial applications [35], which led to its approval for use in humans by the US Food and Drug Administration (FDA) and the European Medicine Agency (EMA).

Our hypothesis is that PLGA nanoparticles can improve MB’s photodynamic effects and contribute to better clinical outcomes in the treatment of chronic periodontitis. Therefore, in the present study, we investigated the effects of aPDT mediated by MB-loaded PLGA nanoparticles (MB-NP) on human dental plaque microorganisms *in vitro* (planktonic and biofilm phase) and *in vivo* (patients with chronic periodontitis).

## 2. Results

### 2.1. In Vitro Studies

In both planktonic and biofilm experiments, groups treated only with free MB, MB-NP or light did not show significant differences compared with the control group (no drug/no light), indicating an absence of toxicity for light, MB, or MB-NP alone (data not shown).

Table 1 summarizes the effects of aPDT on planktonic and biofilm species following their incubation with free MB or MB-NP. Biofilm bacteria showed greater resistance to aPDT treatment than planktonic cells. MB-MN-mediated aPDT was more effective than free MB-mediated aPDT in both planktonic and biofilm phases. MB-MN-mediated aPDT was equally effective on both planktonic and biofilm microorganisms.

**Figure 1:**
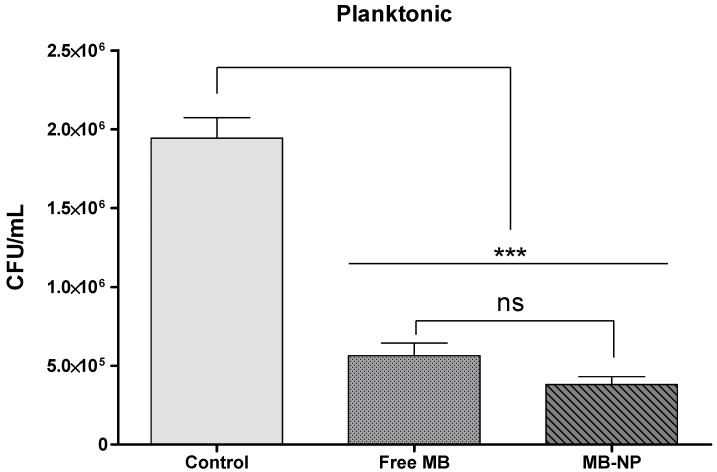
Recovered CFU/mL after antimicrobial photodynamic therapy (aPDT) treatment of planktonic bacteria with free methylene blue (MB) (25 µg/mL) and MB-NP (25 µg/mL equivalent to MB) and visible light at 660 nm with an energy fluence of 20 J/cm^2^. Each bar is the mean values of the means from 10 samples (data from each sample were representative of four independent suspensions). Error bars denote the standard deviation of the mean. The asterisks represent the statistical difference between the groups and the control (one-way ANOVA with Tukey’s *post hoc*). *** *p* < 0.001; ns: not significant; MB: methylene blue; MB-NP: MB-loaded PLGA nanoparticles; CFU: colony forming units.

In the planktonic phase, colony forming units (CFU) levels were reduced by 71% and 80% for MB-mediated and MB-NP-mediated aPDT, respectively (Figure 1). Although MB-NP-mediated aPDT exhibited greater effect on microorganisms in solution compared with free MB, the results were not statistically significant (*p* > 0.05).

**Figure 2:**
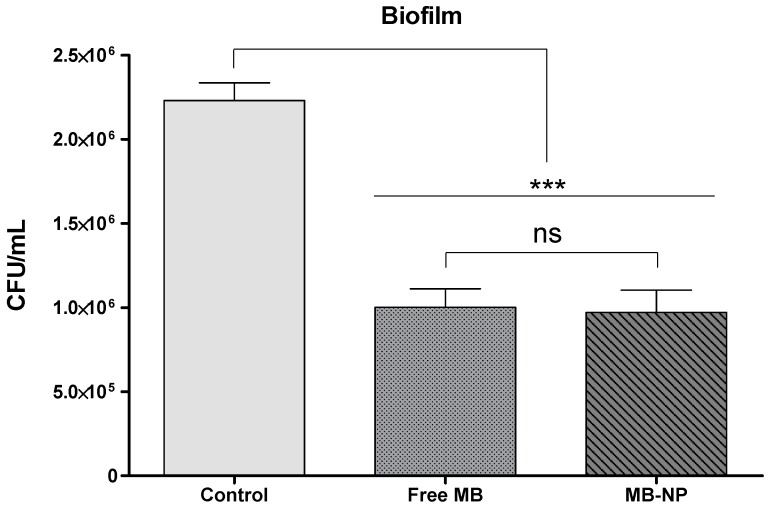
Recovered CFU/mL after aPDT treatment of bacteria growing in biofilms with free MB (25 µg/mL) and MB-NP (25 µg/mL equivalent to MB) and visible light at 660 nm with an energy fluence of 20 J/cm^2^. Each bar is the mean values of the means from 10 samples (data from each sample were representative of four independent biofilms). The asterisks represent the statistical difference between the groups and the control (one-way ANOVA with Tukey’s *post hoc*). *** *p* < 0.001; ns: not significant; MB: methylene blue; MB-NP: MB-loaded PLGA nanoparticles; CFU: colony forming units.

In biofilms, MB-NP-mediated aPDT exhibited 25% greater killing effect compared with free MB (Figure 2). However, differences between the two groups were not statistically significant (*p* > 0.05).

### 2.2. In Vivo Study

**Figure 3:**
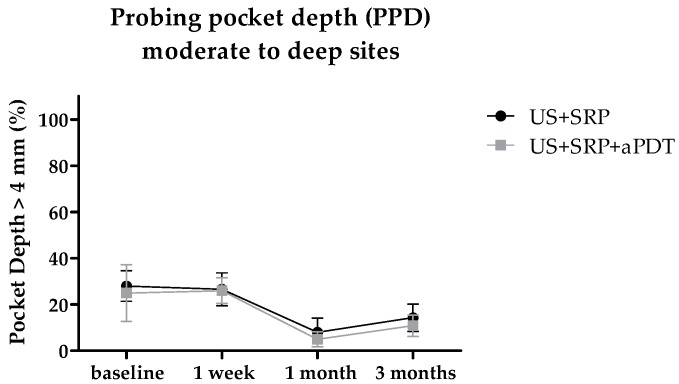
Probing pocket depths. Probing was accessed at baseline, one week, one month, and three months after treatments. Shapes represent mean values from 10 patients at each time point. Error bars represent the standard deviation. US + SRP: ultrasonic scaling and scaling and root planing; US + SRP + aPDT: US + SRP followed by antimicrobial photodynamic therapy.

After treatments, both groups exhibited a trend of a reduction of moderate and deep sites (Figure 3). Results were more evident at one month, with a tendency to return to baseline levels by three months after treatments in both groups. Ultrasonic scaling (US) + SRP associated with aPDT had a slightly better outcome than US + SRP alone (*p* = 0.0298).

**Figure 4:**
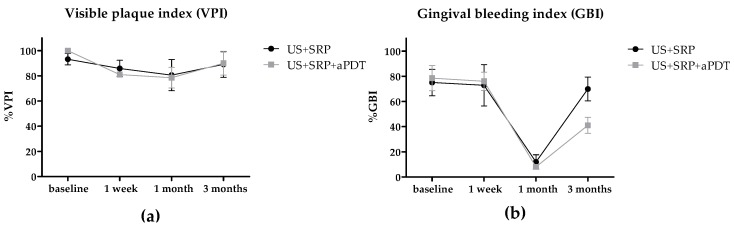
Visible plaque and Gingival bleeding indexes. VPI (**a**) and GBI (**b**) scores were accessed at baseline, one week, one month, and three months after treatments. Shapes represent mean values from 10 patients at each time point. Error bars represent the standard deviation. US + SRP: ultrasonic scaling and scaling and root planing; US + SRP + aPDT: US + SRP followed by antimicrobial photodynamic therapy.

Visible plaque index (VPI) was similar for both groups at all time points, with no statistically significant difference observed (Figure 4a; *p* = 0.9299). After three months, there was a tendency of returning to baseline levels, as noticed for probing pocket depth (PPD). Gingival bleeding index (GBI) percentages decreased drastically and similarly for both groups by one month after treatment (Figure 4b; *p* = 0.4571). Nonetheless, US + SRP + aPDT had a better performance (28.82%) in preventing GBI compared to US + SRP at three months.

**Figure 5:**
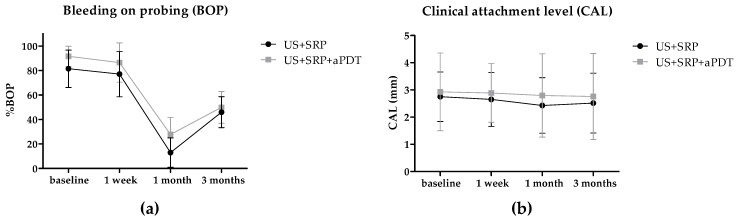
Bleeding on probing and Clinical attachment level. BOP (**a**) and CAL (**b**) scores were accessed at baseline, one week, one month, and three months after treatments. Shapes represent mean values from 10 patients at each time point. Error bars represent the standard deviation. US + SRP: ultrasonic scaling and scaling and root planing; US + SRP + aPDT: US + SRP followed by antimicrobial photodynamic therapy.

The percentage of sites with bleeding on probing (BOP) decreased significantly in both groups one month after treatments, with US + SRP + aPDT group being statistically more effective in reducing BOP than US + SRP (Figure 5a; *p* = 0.0229). Clinical attachment level (CAL) level was sustained in both groups through all time points with no statistical differences between them (Figure 5b; *p* = 0.7826).

Overall, except for CAL, all clinical parameters had an improvement at one month for both treatments.

## 3. Discussion

Recent meta-analyses on the effect of aPDT for periodontitis showed that the use of aPDT as an adjunct to SRP did not yield better results than SRP alone or associated with systemic antibiotics [38] or provided short-term benefits [39] when administrated as a single session. When applied in multiple sessions, however, aPDT has been proven safe and effective as an adjunctive therapy in periodontal disease treatment, as evidenced by a plethora of studies [10,11,12,13]. Several antimicrobial resistance mechanisms may be responsible for the reduced susceptibility of dental plaque to aPDT. These include the expression of certain phenotypes by biofilm species [40], the slow growing or starved state of microorganisms within biofilms [41], the inactivation of PS [42], the presence of multidrug resistance pumps in bacterial cells that expel the PS [43], and the restricted penetration of PS in oral biofilms [44]. The treatment of biofilm-associated bacterial infections poses challenges due to several antimicrobial resistance mechanisms of biofilms [45]. Possible explanations for the reduced susceptibility of dental plaque to aPDT include one way to overcome the incomplete eradication of dental plaque microorganisms is to develop a delivery system that significantly improves the pharmacological characteristics of the PS.

In the present study, our hypothesis was that MB-loaded PLGA nanoparticles would exhibit a superior photodynamic effect on human dental plaque bacteria—in planktonic and biofilm phase—compared with free MB. Additionally, a pilot study was conducted with 10 patients to evaluate the efficacy of aPDT with MB-NP on chronic periodontitis as an adjunct to ultrasonic scaling and SRP. This is the first *in vivo* study that employed the use of polymeric nanoparticles as carriers of PS for aPDT. PLGA nanocarriers have been used successfully in drug delivery of MB *in vitro*, previously [33,46]. MB lacks its photochemical properties when it is encapsulated in PLGA and regains its phototoxicity when it is released by PLGA [47].

In suspensions, the synergism of light and MB-NP showed a greater killing effect (80.5%) over free MB (71%). In oral microcosm laboratory biofilms, nanoparticles and free MB reduced bacterial viability by 79% and 55%, respectively. Although, differences between aPDT groups in both planktonic and biofilm phase were not statistically significant, photodynamic killing results were similar in all experiments. The greater photodynamic effect of MB-loaded nanoparticles over free MB in suspensions and biofilms was also demonstrated by Klepac-Ceraj *et al.* (2011) [46]. However, in the present study the effect of aPDT on both planktonic and biofilm microorganisms was almost the same; 80.5% *vs.* 79%, respectively. These data show that nanoparticles were able to penetrate the biofilm and target microorganisms rapidly. Our findings are supported by recent studies that have demonstrated that nanoparticles, regardless their composition, can successfully disrupt the biofilm matrix, allowing for a deeper penetration and a sustained release of drugs [48,49,50,51], as well as increasing drug stability and retention [52,53,54,55].

Our clinical pilot study clearly demonstrated the safety of aPDT. No adverse effects were reported. In this study, the effect of aPDT as an adjunct to US and SRP was compared to US and SRP alone. All clinical parameters (VPI, GBI, BOP, and PPD) in both groups showed the greatest improvement one month following treatment with the exception of CAL that was sustained in both groups through all time points. After one month, all parameters showed a similar increasing trend. At all time points, there were no statistically significant differences between the two treatment groups. However, at three months US + SRP + aPDT showed a greater effect (28.82%) on GBI compared to US + SRP.

At three months after treatment all clinical parameters started to return to baseline levels, for both US + SRP and US + SRP + aPDT, which indicates bacterial recolonization of periodontal pockets. Treatment rebound due to bacterial recolonization is a common feature of chronic periodontitis treatments, regardless the technique employed, as evidenced by studies of Petersilka [56], Zijnge [57], Teles [58], and Sanz-Sánchez [59]. However, the studies of Novaes Jr [60] and Petelin [61] demonstrate that different groups of bacteria are affected after treatment with aPDT or SRP, resulting in a distinct pattern of recolonization. In fact, aPDT was more effective in reducing the presence of Red Complex species, such as *Tanerella forsythia* and *Treponema denticola*, and *Aggregatibacter actinomycetemcomitans*, a species known by its association with localized aggressive periodontitis [60,61]. Taken together, those findings highlight that the association of classical SRP to aPDT in the treatment of periodontitis sums up benefits.

Our findings suggest that MB-NP have the potential to be used as carriers of MB for photodynamic inactivation of dental plaque bacteria. MB-NP and light exhibited a greater killing in biofilms. Our hypothesis is that MB-NP were able to diffuse and released MB within biofilms. This may not be the case in the clinical pilot study that comprised a small number of patients, and, therefore, restricts any broader conclusions. Future studies should define the appropriate aPDT dosimetry (MB concentration, incubation time, power density, and energy fluence) for effective elimination of biofilm species. The possibility of multiple applications of aPDT should also be explored. These improvements and changes in the treatment protocol may demonstrate the adjunctive benefit of aPDT in periodontitis.

## 4. Materials and Methods

### 4.1. Subjects and Samples

Forty-seven patients were analyzed, 27 were excluded due to one or more exclusion criteria, and 20 patients entered the study. Ten patients were assigned to *in vitro* assays and ten patients participated in the *in vivo* study. All subjects gave their informed written consent to donate dental samples for inclusion before they participated in the study. The study was conducted at the Dental Office of University of Sao Paulo (Optics Group—Instituto de Fisica de Sao Carlos, Sao Carlos, SP 15980-900, Brazil), and conducted in accordance with the Declaration of Helsinki, and the protocol was approved by the Dental School Research Ethics Committee at Araraquara, UNESP Univ. Estadual Paulista (Protocol #04/11). The *in vivo* study was approved by Human Research Committee–Process HCRP n° 1857/2008. Patients completed a health history questionnaire to ensure that they were medically qualified for participation in the study. Inclusion criteria for the study were patients diagnosed with chronic periodontitis [62] who were no smokers, had at least four teeth in each quadrant (16 teeth on their functional dentition, excluding third molars) and had, at least, two posterior teeth with pocket depth ≥4 mm and bleeding on probing (BOP). The exclusion criteria were: smokers, orthodontic brackets, pregnancy, diabetes mellitus, use of anti-inflammatory or antibiotic agents within the previous three months, periodontal therapy during the six months prior to sampling, or use of any medications associated with the gingival disease. The deepest pockets (>5 mm) of each quadrant were used for plaque sampling after the photodynamic and/or periodontal procedure.

### 4.2. In Vitro Study—Sample Collection

Using individual sterile Gracey curettes, dental plaque samples from subgingival sites were taken in each subject (five to eight samples per subject; pockets >5 mm) and placed immediately into pre-reduced, anaerobically sterilized Ringer’s solution (Anaerobe Systems, Morgan Hill, CA, USA), forming a sample pool. Bacteria from the plaque samples were dispersed by sonication and homogenezation through Pasteur pipettes. The optical density of the bacterial suspensions was measured in a spectrophotometer and then the pool sample from the ten subjects was divided into two parts, for planktonic and biofilm assays.

### 4.3. Preparation of Enriched Agar Plates

Blood agar enriched with hemine, menadione, and *N*-acetylmuramic acid was prepared. Medium composition comprised 5% defibrinated sheep blood (NewProv LTDA, Pinhais, PR, Brasil), 2.6% brain heart infusion agar (BHI—Difco Laboratories, Detroit, MI, USA), 2% trypticase soy agar (TSA—Difco (Franklin Lakes, NJ, USA)), 1% yeast extract (BBL, Cockeysville, MD, USA), 1% hemine (Sigma Chemical Co., St. Louis, MO, USA), 1% *N*-acetylmuramic acid (Sigma Chemical Co.), and 0.5% menadione (Sigma Chemical Co.). Agar mixture was sterilized in autoclave and, after cooling it to 50 °C, sheep blood, menadione, and *N*-acetylmuramic acid were added to the mixture under aseptic conditions. Medium was then dispensed into 96-well plates (TPP, Zellkultur testplatte, Trasadingen, Switzerland), 150 µL per well, and allowed to solidify. Plates were stored at 4 °C.

### 4.4. Development of Plaque-Derived Biofilms

For biofilm development, the bacterial inoculum (in BHI broth) was adjusted to contain approximately 10^7^ cells/mL. Approximately 1.5 × 10^6^ bacteria (150 µL) were dispensed into a blood agar well. For each experimental group 4 wells were used. Plates were incubated at 35 °C in anaerobic atmosphere (80% N_2_, 10% H_2_, and 10% CO_2_) for seven days. At day 2 the broth was carefully aspirated and fresh BHI broth was added to each well. Then, fresh BHI broth was added daily to each well, very slowly, to avoid disruption of the biofilm.

### 4.5. Photodynamic Treatment in Vitro

A diode laser with a central wavelength of 660 nm coupled to a 1 mm optical fiber that delivered light into a lens was used for both planktonic and biofilm studies. The system formed a uniform circular spot, 2 cm in diameter, which was able to irradiate a group of four wells in a 96-well plate each time, from above, at room temperature in the absence of surrounding light. The power density was measured using a powermeter. For both planktonic and biofilm experiments power density was 100 mW/cm^2^ and energy fluence was 20 J/cm^2^. Microorganisms in planktonic and biofilm phase were exposed only once to light. Free MB concentration was 25 μg/mL and the final concentration of MB-NP was 25 μg/mL equivalent to MB. Experimental groups were: (1) no light/no PS (CONTROL); (2) treated only with free MB; (3) treated only with MB-NP; (4) treated only with light; (5) treated with light and free MB; and (6) treated with light and MB-NP.

#### 4.5.1. Planktonic Bacteria

Aliquots of bacterial suspensions (10^8^ cells/mL) were placed in sterile microtubes and centrifuged at 7000 rpm for 4 min. One milliliter of sterile free MB or MB-NP was then added after discarding the supernatants. Bacterial cells were suspended in free MB or MB-NP and placed in four wells of 96-well plates for 10 min before exposure to light. Following aPDT, bacterial suspensions underwent serial dilutions in BHI broth, and 100 µL aliquots were plated on blood agar and incubated under anaerobic conditions at 35 °C for seven days prior to CFU scoring.

#### 4.5.2. Biofilms

Carefully, growth medium was aspirated from each well of 96-well plates and replaced by 150 µL of sterile free MB or MB-NP. Biofilms were then incubated for 10 min followed by exposure to light. After aPDT, bacteria from each well were gently scraped using a sterile bacteriological loop, dispersed in BHI broth and measured in a spectrophotometer at 600 nm. After, serial dilutions were prepared and 100 µL aliquots were plated on blood agar plates, which were incubated anaerobically at 35 °C for seven days prior to CFU counting.

### 4.6. Preparation and Characterization of PLGA Nanocarriers

**Figure 6:**
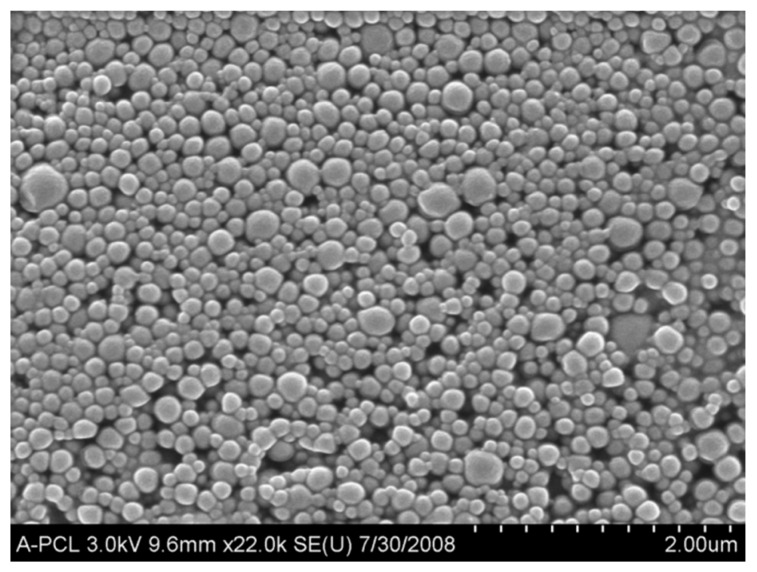
Scanning electron micrograph (SEM) of PLGA nanoparticles. Figure shows an SEM image of higher magnification with spherical nanoparticles of 150–250 nm in diameter.

MB loaded into PLGA nanoparticles (10% *w*/*w*) were prepared in the Department of Pharmaceutical Sciences at Northeastern University as previously described [33]. Briefly, a PLGA (76 mg) and Pluronic^®^ F-108 (14 mg) solution was prepared in 5 mL of acetone. MB as oleate salt (Sigma Chemicals Co.) was dissolved at 10% (*w*/*w*) concentration in the PLGA acetone solution for the preparation of the MB-loaded nanoparticles. To insure that the formed nanoparticles have a stable hydrophilic surface, which resists aggregation, pluronic triblock copolymers were added to the polymer solution in acetone at 20% (*w*/*w*). The acetone solution was added into an aqueous (50 mL) solution under vigorous stirring and left to stir overnight. The following day, nanoparticles were centrifuged at 10,000 rpm for 20 min, washed twice with deionized distilled water and lyophilized. Data regarding nanoparticle characterization were previously published [33]. Figure 6 shows a scanning electron micrograph of blank PLGA nanoparticles.

### 4.7. In Vivo Study

This 3-month study evaluated clinically the effectiveness of the adjunctive use of polymeric nanoparticle-based aPDT following periodontal instrumentation with ultrasonic scaling and mechanical scaling and root planing (SRP) in patients (seven men; 13 women; aged 20–70) with moderate to advanced chronic periodontitis [62]. All enroled patients completed the study. The study investigated the correlation of the clinical parameters before and after aPDT treatment in periodontitis sites in the same patient, following a split-mouth design. All four quadrants received treatment. Two of them (one lower and one upper jaw) received non-PDT ultrasonic scaling followed by mechanical SRP with Gracey curettes and the other two quadrants received ultrasonic scaling (US) and mechanical SRP followed by aPDT. Prior to aPDT, MB-NP were applied as a mouthwash (MB-NP dispersed in PBS 1×) and then periodontal pockets were irrigated with the same PS solution for 10 min. PDT was applied as a single session. The effect of the two different treatment groups—US + SRP *vs.* US + SRP + aPDT—was investigated on clinical parameters such as probing pocket depth (PPD), visible plaque index (VPI), gingival bleeding index (GBI), bleeding on probing (BOP), and clinical attachment level (CAL). All clinical parameters measured at baseline, one week, one month, and three months, and recorded by a single examiner. Oral hygiene procedures were instructed and reinforced at every appointment. PPD at baseline was divided into two categories: shallow sites (pocket depth from 1 to 3 mm) and moderate to deep sites (moderate: 4–6 mm; deep ≥ 7 mm).

### 4.8. Data Collection—Measurement Reproducibility

Calibration trials were performed prior to the study to ensure adequate intra-examiner reproducibility (kappa statistic ≥ 90%). Intra-examiner kappa values were 0.97 (PPD) and 0.93 (CAL). All measurements were performed by a single examiner using a standard University of North Carolina probe with millimeter markings.

### 4.9. Clinical Parameters

Clinical parameters that were examined in this study included presence or absence of visible plaque index (VPI), gingival bleeding index (GBI), and bleeding on probing (BOP). Full-mouth probing pocket depth (PPD) and clinical attachment level (CAL) were measured by a North Carolina manual periodontal probe (North Caroline Probe, Hu-Friedy, Chicago, IL, USA) at six sites per tooth in all teeth except third molars, at baseline, one week, one month, and three months after the aPDT and non-aPDT associated with ultrasonic scaling and periodontal treatment (US-SRP).

### 4.10. Statistical Analysis

*In vitro* data were expressed as the mean plus standard deviation (SD) and were analyzed by one-way ANOVA with Tukey’s *post hoc* test using GraphPad Prism^®^ Version 5.01 software (GraphPad Software Inc., La Jolla, CA, USA). Differences were considered to be significant when *p* < 0.05 (confidence level of 95%). For *in vivo* data, differences between groups were sought using the repeated measures *t*-test, also using GraphPad Prism^®^ Version 5.01 software. Differences with a *p*-value <0.05 at a confidence level of 95% were considered significant.

## Figures and Tables

**Table 1:** Effects of antimicrobial photodynamic therapy (aPDT) on planktonic and biofilm species *in vitro*.

Planktonic	Free MB	MB-NP
log_10_ steps reduction	0.58	0.71
% reduction	70.97	80.40
CFU/mL average	564,167	380,833
log_10_ average	5.70	5.58
SD	0.21	0.20
**Biofilm**	**Free MB**	**MB-NP**
log_10_ steps reduction	0.35	0.69
% reduction	55.04	79.37
CFU/mL average	1,002,500	972,500
log_10_ average	6.00	5.99
SD	0.17	0.22

Subgingival plaque samples from 10 patients were assayed either in planktonic phase or biofilms (*n* = 12 replicates). Experimental groups (control, MB in solution, and MB in PLGA nanoparticles) were compared via one-way ANOVA followed by Tukey’s *post hoc*. MB: methylene blue; SD: standard deviation; CFU: colony forming units.

## Abbreviations

aPDT	antimicrobial photodynamic therapy
BOP	bleeding on probing
CFU	colony-forming unity
CAL	clinical attachment level
GBI	gingival bleeding index
MB	methylene blue
MB-NP	methylene blue-loaded PLGA nanoparticles
NP	nanoparticle
PLGA	poly(Lactic-*co*-Glycolic Acid)
PPD	probing pocket depth
ROS	reactive oxygen species
SRP	scaling and root planing
VPI	visible plaque index
